# A Cellulose/Chitosan Dual Cross‐Linked Multifunctional and Resilient Hydrogel for Emergent Open Wound Management

**DOI:** 10.1002/adhm.202304676

**Published:** 2024-02-13

**Authors:** Shengchang Lu, Hui Wu, Shengbo Ge, Liulian Huang, Lihui Chen, Chris Connor, Zhanhu Guo, Yunhong Jiang, Ben Bin Xu, Wanxi Peng

**Affiliations:** ^1^ School of Forestry Henan Agricultural University Zhengzhou 450002 P. R. China; ^2^ College of Material Engineering Fujian Agriculture and Forestry University Fuzhou Fujian 350002 P. R. China; ^3^ National Forestry and Grassland Administration Key Laboratory of Plant Fiber Functional Materials Fuzhou Fujian 350002 P. R. China; ^4^ Co‐Innovation Center of Efficient Processing and Utilization of Forest Resources College of Materials Science and Engineering Nanjing Forestry University Nanjing 210037 P. R. China; ^5^ Mechanical and Construction Engineering Northumbria University Newcastle Upon Tyne NE1 8ST UK; ^6^ Hub for Biotechnology in the Built Environment Department of Applied Sciences Northumbria University Newcastle upon Tyne NE1 8ST UK

**Keywords:** adhesive, cellulose, chitosan, first‐aid tape, hybrid hydrogel

## Abstract

Adhesive hydrogel holds huge potential in biomedical applications, such as hemostasis and emergent wound management during outpatient treatment or surgery. However, most adhesive hydrogels underperform to offer robust adhesions on the wet tissue, increasing the risk of hemorrhage and reducing the fault tolerance of surgery. To address this issue, this work develops a polysaccharide‐based bioadhesive hydrogel tape (ACAN) consisting of dual cross‐linking of allyl cellulose (AC) and carboxymethyl chitosan (CMCS). The hygroscopicity of AC and CMCS networks enables ACAN to remove interfacial water from the tissue surface and initializes a physical cross‐link instantly. Subsequently, covalent cross‐links are developed with amine moieties to sustain long‐term and robust adhesion. The dual cross‐linked ACAN also has good cytocompatibility with controllable mechanical properties matching to the tissue, where the addition of CMCS provides remarkable antibacterial properties and hemostatic capability. Moreover, compared with commercially available 3 M film, ACAN provides an ultrafast wound healing on tissue. The ACAN hybrid hydrogels have advantages such as biocompatibility and antibacterial, hemostatic, and wound healing properties, shedding new light on first‐aid tape design and advancing the cellulose‐based materials technology for high‐performance biomedical applications.

## Graphical abstract

A first‐aid hydrogel tape is fabricated via dual cross‐linking design strategy under light initiation based on cellulose and chitosan, enabling potential multifunctionality for emergent open wound management. The obtained ACAN hydrogel tape possesses tissue‐adaptive mechanical performance, robust tissue adhesion, antibacterial, rapid hemostasis, and wound closure properties, making it a promising candidate for first‐aid applications.

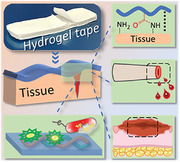

## 1 Introduction

First‐aid products play critical roles in emergent healthcare services and disaster incidents by effectively providing wound management.^[^ ^1^ ^]^ Among these products, sutures and staples serve as mainstays for wound management and cease bleeding in clinical treatment.^[^ ^2^ ^]^ There is a consistent demand to develop alternative first‐aid products with more user‐friendly approach to manage the wound at the absence of sutures and staples. With this regard, several biomedical materials have emerged such as fibrin glue (Tisseel), collagen‐based (Angio‐Seal), and cyanoacrylate‐based (Histoacryl) adhesives, which usually show one or more disadvantages from weak mechanical properties, poor adhesion strength, or toxicity concerns.^[^ ^3^ ^]^ Hydrogel, a promising candidate for wound dressings, has been developed as an antibacterial barrier, hemostatic sealing, and other tissue engineering applications.^[^ ^4^ ^]^ Hydrogel‐based bioadhesives can be processed with different methods and mechanisms,^[^ ^5^ ^]^ such as fast gelation based on dynamic covalent bonds^[^ ^6^ ^]^ or light‐activated techniques,^[^ ^7^ ^]^ block ex‐situ hydrogels,^[^ ^8^ ^]^ and hydrogel‐based patches or films.^[^ ^9^ ^]^ Compared to medical goods, hydrogels with unique structure–property relationship endow them adaptive mechanical properties, excellent biocompatibility, on‐demand degradability, and strong adhesion to substrates, enabling a versatile platform for subsequent integration into biomedical devices and systems.^[^ ^3^, ^10^ ^]^ However, a few technical challenges, such as a short gelation time, nontoxic initiators/cross‐linkers, and antimicrobial or user‐friendly properties, must be met prior to the scaling up production.

As globally abundant natural polysaccharides, cellulose and chitosan have enormous potential in biomedical applications for their biocompatibility, low cost, and ecofriendliness.^[^ ^11^ ^]^ Even the cellulose has explicit technical disadvantage in biomedical application for its insolubility in water,^[^ ^12^ ^]^ some progresses have been made such as carboxymethyl cellulose/ε‐polylysine hydrogel for wound repair,^[^ ^13^ ^]^ polyacrylamide/bacterial cellulose hydrogel dressings,^[^ ^14^ ^]^ and cellulose‐based adhesive hydrogel for hemostasis and wound healing.^[^ ^15^ ^]^ Chitosan and its derivatives based functional hydrogels have demonstrated excellent biocompatibility and antibacterial feature for several biomedical scenarios, such as quaternized chitosan‐containing antibacterial hydrogel, carboxymethyl chitosan (CMCS) hydrogel dressing,^[^ ^16^ ^]^ and aldehyde‐modified cellulose/catechol‐conjugated chitosan for bone regeneration.^[^ ^17^ ^]^ Meanwhile, it is worth noting that the abovementioned materials have explicit limitations, such as a lack of hemostatic capacity and weak mechanical/adhesion strength, which prevent their application as a first‐aid product.

To achieve robust adhesion for hemostasis, *N*‐hydroxysuccinimide (NHS) esters have been widely used in bioadhesive because of the amide bonds formed with primary amines on the tissue surface.^[^ ^3a^ ^]^ A representative example is the poly(acrylic acid) (PAA) grafted with NHS ester groups,^[^ ^2^, ^18^ ^]^ which endow the adhesive tape with outstanding adhesion for wet tissue and device. Some NHS ester‐based polymers have been developed for peripheral nerve regeneration, hemostasis, and wound healing.^[^ ^9^, ^19^ ^]^ However, there are limited reports on grafting NHS ester moieties onto cellulose chains to enable a new group of adhesive materials for biomedical application.

Herein, we develop an adhesive hydrogel tape for effective wound management by introducing a dual cross‐linking network based on allyl cellulose (AC), CMCS, and acrylic acid NHS ester (AA‐NHS), to significantly improve the tissue adhesion and hemostatic capacity of cellulose‐based hydrogels. The obtained AC and CMCS polymers possess excellent water solubility via an etherification reaction, as well as polymerizable ability for AC, which overcomes the solubility challenges of cellulose and chitosan in the preparation of biomedical hydrogels in homogeneous systems. The cooperation effect of interfacial water removal, physical cross‐linking, and chemical cross‐linking confers the hydrogel tapes with long‐term and robust adhesion to wet tissue surfaces. With physical sealing and blood coagulation effect, the hydrogel tapes exhibit desirable hemostatic performance. Simultaneously, the obtained hydrogel exhibits remarkable cytocompatibility and antibacterial activity without the use of additional antibiotics owing to the inherent nature of CMCS. The dual cross‐linked cellulose/chitosan hydrogel tape possesses robust adhesion, antibacterial, hemostasis, and wound healing properties, making it a potential biomaterial for first‐aid applications.

## 2 Results and Discussion

### 2.1 Design Strategy for Hydrogel Tapes

**Figure 1:**
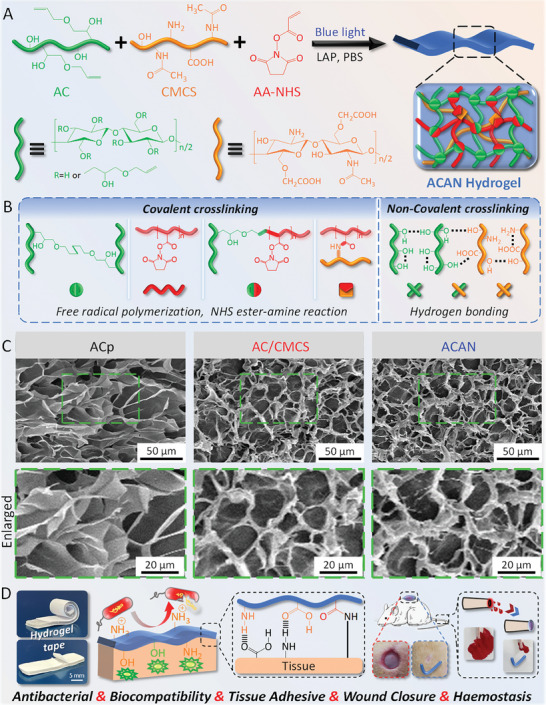
Design strategy of the ACAN hydrogel tape. A,B) Illustration of the hydrogel preparation and dual cross‐linked network structure. C) Microscopic morphology of the hydrogels. D) Schematic of the potential multifunctional application of hydrogel tape.

The multifunctional ACAN hydrogels are synthesized by incorporating CMCS and AA‐NHS into the AC network (**Figure** **1**A) via a light‐activated strategy. The water‐soluble AC, CMCS, and AA‐NHS are synthesized with unsaturated double bonds (AC and AA‐NHS) and carboxyl groups (CMCS) while retaining the functionality of hydroxyl, amino, and succinimide groups. The presence of double bonds in AC and AA‐NHS is confirmed by ^1^H nuclear magnetic resonance (NMR) spectroscopy (Figure S1A,C, Supporting Information), which imparts capability to AC and AA‐NHS for free radical polymerization and the potential for chemical cross‐linking. To obtain the CMCS polymer, carboxyl groups are introduced to substitute the hydroxyl group on the *O*‐position at C3/C6, retaining the primary amino group in chitosan with a content of 0.63 ± 0.06 mmol g^−1^. The degree of carboxymethyl substitution of CMCS is 0.61, as being calculated from the NMR spectra in Figure S1B, Supporting Information.

Lithium phenyl‐2,4,6‐trimethylbenzoylphosphinate (LAP) is a nontoxic photoinitiator that provides biosafety for polymerization reactions.^[^ ^20^ ^]^ The diagram in Figure 1B illustrates the construction process of dual cross‐linked network, where AC and AA‐NHS form homopolymers or copolymers via free radical polymerization, and AA‐NHS conjugates with CMCS via the NHS ester‐amine reaction. In addition to chemical cross‐linking, a physical cross‐linking network based on hydrogen bonding interactions is initiated due to the residual hydroxy groups on AC chains and the abundant hydroxyl, carboxy, and amino groups on CMCS chains.^[^ ^21^ ^]^ The scanning electron microscopy (SEM) images in Figure 1C show 3D microporous structures with pore sizes of ≈20 µm in AC/CMCS (containing LAP, AC, and CMCS) and ACAN (containing LAP, AC, CMCS, and AA‐NHS) hydrogels, which are denser than ACp (containing LAP and pure AC) hydrogel. Furthermore, ATR‐FTIR (Figure S2, Supporting Information) results demonstrate that chemical cross‐linking occurs via free radical polymerization. The degree of cross‐linking (*D*_c_) of ACp, AC/CMCS, and ACAN hydrogels is approximately 97% (Table S1, Supporting Information) which indicates that the as‐prepared hydrogel tapes are almost completely cross‐linked with very low amount of free monomers or polymers with low molecular weight. Compared to the ACp hydrogel, the addition of CMCS and AA‐NHS increases the solid content and component by generating denser structures and rougher textures, which facilitate the multifunctionality (Figure 1D) for potential biomedical application.

### 2.2 Mechanical, Swelling, and Adhesion Performances

**Figure 2:**
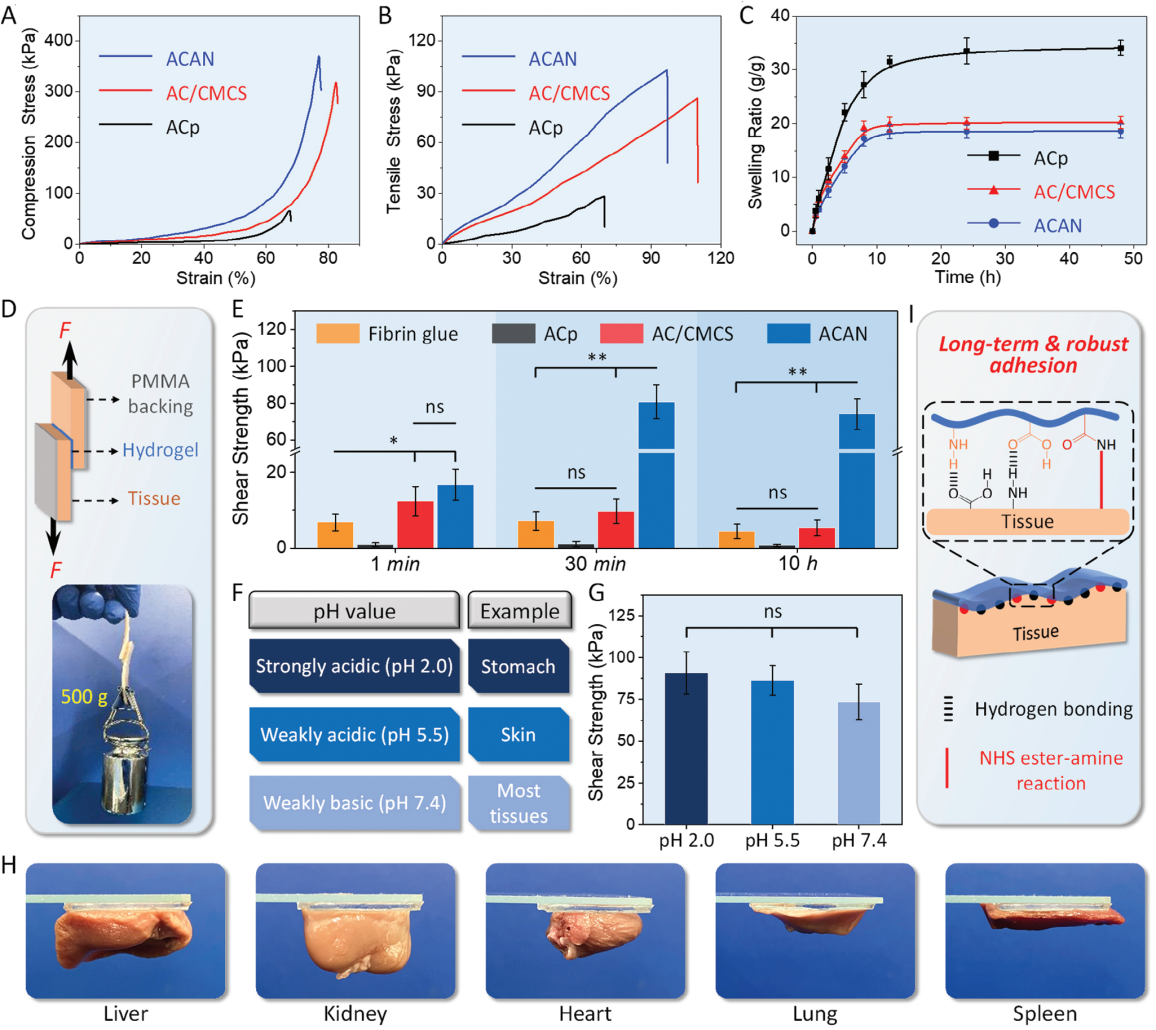
Characterization of mechanical and adhesive properties: A) compression and B) tensile stress–strain curves of the hydrogels. C) Swelling ratios of the hydrogels. D) Illustration of the lap shear test. The insert reveals that the hydrogel tape‐adhered porcine skin can withstand a weight of 500 g. E) Maximum shear strength of various hydrogel tapes and Fibrin glue on porcine skin for different durations. F) Various pH values of the tissue. G) Maximum shear strength of the ACAN hydrogel tape on porcine skin at different pH values. H) Adhesion performance of the ACAN hydrogel tape on various rat tissues. I) Mechanisms of adhesion of the ACAN hydrogel tape on skin tissue. (Error bars indicate standard error of the means, *n* = 5; ns, not significant; * indicates significant difference, ^*^ *p* < 0.05, ^**^ *p* < 0.01, ^***^ *p* < 0.001).

The ACp hydrogel shows a compressive stress of 67.6 ± 17.8 kPa with a maximum strain of <70% (**Figure** **2**A and Figure S3A, Supporting Information). By adding more CMCS, the compressive stress of AC/CMCS reaches 325.1 ± 28.4 kPa, with a maximum compressive strain ≥75%. Furthermore, the compressive stress of ACAN increases to 380.1 ± 20.3 kPa following the addition of AA‐NHS. In the tensile test (Figure 2B and Figure S3B, Supporting Information), the ACp hydrogel obtain a stress of 28.2 ± 9.2 kPa with a maximum elongation of 70%. With the addition of CMCS, the tensile stress of AC/CMCS gel increases to 86.3 ± 10.8 kPa with a maximum tensile strain of 113.3% ± 15.1%. Furthermore, ACAN has a tensile stress of 102.8 ± 28.4 kPa with a slight reduction in tensile strain (106.2% ± 13.2%). Compared to ACp hydrogels, the addition of CMCS effectively enhances the mechanical properties of AC/CMCS hydrogel. In addition, the polymerization of AC and AA‐NHS (Figure S2, Supporting Information), the potential NHS ester‐amine reaction,^[^ ^3a^ ^]^ and an essentially fully cross‐linked structure (*D*_c_ = 97.4±0.3%) further improve the tensile and compressive stress of ACAN hydrogels. The compression moduli of AC/CMCS and ACAN increase to 45.6 ± 7.4 and 53.1 ± 8.2 kPa, respectively, compared with that of ACp hydrogel (19.5 ± 6.8 kPa, Figure S3C,D, Supporting Information). The elastic moduli of AC/CMCS and ACAN hydrogels are 78.8 ± 9.7 and 95.3 ± 12.7 kPa, respectively, higher than that of ACp hydrogel (28.7 ± 8.1 kPa), after adding CMCS and AA‐NHS. Figure S3E,F in Supporting Information shows a significant increase in work of fracture for the hybrid hydrogels, by adding CMCS and AA‐NHS. In addition, the elastic modulus of ACAN hydrogel (≈100 kPa, Figure S3G, Supporting Information) provides a good match for majority of tissues, except cartilage (≈100 MPa) and bone (≈1 GPa).^[^ ^3^, ^22^ ^]^

The swelling ratio of hydrogel is another critical factor closely dependent on the cross‐linking and overall network architecture. In Figure 2C, ACp hydrogel tape presents a swelling ratio of 34.2 ± 1.4 g g^−1^ when it achieves adsorption equilibrium after 48 h. The swelling ratios of AC/CMCS and ACAN are 20.1 ± 1.1 and 18.6 ± 1.2 g g^−1^, respectively, when it reaches equilibrium state. The addition of CMCS and polymerization of AA‐NHS increases the solid content of hydrogels and contributes to the formation of denser structure with cross‐linked network. It promotes a faster swelling equilibrium while avoids the impairment of mechanical properties for hydrogel by the water absorption.^[^ ^23^ ^]^

We next perform an enzymatic degradation experiment (Figure S3A, Supporting Information) to evaluate the degradation behavior of samples.^[^ ^24^ ^]^ A remaining mass of 83.8% ± 4.2% is obtained for the ACp hydrogel after 30 days of incubation. With the addition of CMCS and AA‐NHS polymer, the remaining mass of AC/CMCS and ACAN hydrogels decrease to 24.8% ± 3.1% and 18.9% ± 4.1%, respectively. Notably, AC/CMCS and ACAN hydrogels rapidly degrade in the first 15 days, then gradually settles. As for the degradation in phosphate‐buffered saline (PBS) without the lysozyme solution, all samples remain at >75% of their initial mass (Figure S4B, Supporting Information). The hypothesis for this phenomenon is that lysozyme selectively hydrolyzes *β*−1,4 glycosidic linkages in CMCS chains but not cellulose. Moreover, as an important immune factor, lysozyme is widely distributed in tissues.^[^ ^25^ ^]^ Thus, enzymatic degradation of AC/CMCS and ACAN not only overcome the barrier of limited degradation of cellulose‐based hydrogels^[^ ^15^ ^]^ but also allow for the development of materials with controllable degradation or programmable structures for biomedical applications.

A typical lap shear test is conducted by using porcine skin as the reference tissue, the ACAN tape‐adhered tissue withstand a weight of 500 g (Figure 2D). Figure 2E shows that ACAN and AC/CMCS tapes have shear strengths of 16.7 and 12.3 kPa, respectively, after 1 min of deployment on skin, which is approximately twice that of commercially available fibrin glue (*p* < 0.05). Interestingly, the shear strength of ACAN tape increases to 80.8 ± 9.2 kPa after 30 min of deployment, which is significantly higher than that of AC/CMCS tape and fibrin glue (*p* < 0.01). Over a period of 10 h, the shear strength of ACAN remains over 70 kPa, whereas that of AC/CMCS and fibrin glue barely exceed 10 kPa. Obviously, the reduced shear strength of AC/CMCS tapes is caused by the instability of intermolecular bonds under wet conditions.^[^ ^26^ ^]^ Thus, the addition of CMCS improves the shear strength and further polymerization of AA‐NHS on this basis greatly enhances the long‐term and high‐strength adhesion of the of ACp tapes.

Next, we examined the tensile strength and interfacial toughness of fibrin glue and hydrogel tapes. In Figure S5B, Supporting Information, the ACAN tape has a tensile strength of >90 kPa after adhering to the skin for 30 min, which is significantly stronger than that of fibrin glue and AC/CMCS tape (*p* < 0.001). To verify the effect of hydrogel composition on interfacial toughness, a 180° peel test on porcine skin is conducted (Figure S5C, Supporting Information). The results demonstrate that ACAN tape possesses high interfacial toughness (≈170.1 J m^−2^) and significantly outperforms fibrin glue (≈13.3 J m^−2^, *p* < 0.001). In Figure S5D,E (Supporting Information) the burst pressure of ACAN tape reaches 126.7 ± 11.3 mm Hg after 1 min of application and increased to 218.2 ± 16.4 mm Hg, remarkably higher than that of the fibrin glue. Notably, the burst pressure of ACAN tape satisfies the normal systolic blood pressure (120 mm Hg). Moreover, the ACAN tape establishes strong adhesion with a shear strength >70 kPa, even under different physiological pH (Figure 2F,G). We demonstrate to firmly adhere the ACAN tapes on the surface of different rat organs (Figure 2H). The potential mechanism for the hydrogel‐tissue adhesion is illustrated in Figure 2I. Apart from the hydrogen bonding formed by interfacial carboxyl, amino, and hydroxyl groups,^[^ ^16^ ^]^ the ACAN hydrogel can cleave the NHS ester moieties and then covalently cross‐linked with the primary amino group from tissue,^[^ ^19b^ ^]^ resulting in superior adhesion than AC and AC/CMCS hydrogels.

### 2.3 In Vitro Antibacterial Activity, Cytocompatibility, and Hemostatic Capability Tests

**Figure 3:**
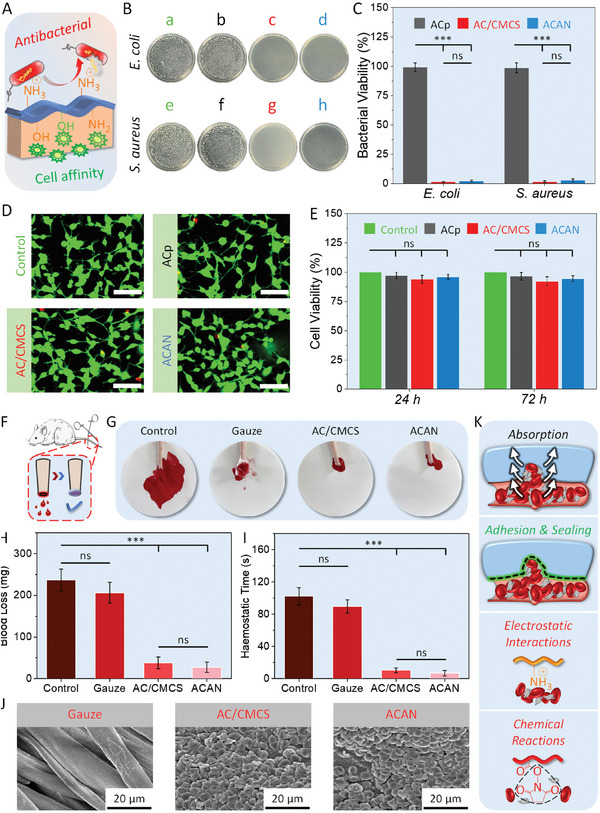
Assessment of bio‐properties. A) Illustration of the antibacterial and cytocompatible properties of the ACAN hydrogel tape. B) Photographs of bacterial colony: Control group (a,e), ACp (b,f), allyl cellulose (AC) and carboxymethyl chitosan (CMCS) (c,g), and ACAN (d,h) hydrogels against different bacteria culture. C) Bacterial viability for *Escherichia coli* and *Staphylococcus aureus* treated with different hydrogels. D) Live–dead staining of NIH‐3T3 cells cultured on various hydrogels for 72 h (scale bar: 100 µm). E) Cell viability of NIH‐3T3 cells using the CCK‐8 assay cultured with various hydrogels after 24 and 72 h. F) Illustration of the rat tail amputation hemostatic model. G) Images of rat tail hemostasis treated with a blank control, medical gauze, AC/CMCS, and ACAN hydrogel tapes. H) Assessment of maximum blood loss and I) hemostatic time for the rat tail amputation model in various treatment groups. J) Scanning electron microscopy images of blood cells attached to gauze, AC/CMCS, and ACAN surfaces. K) Diagram showing the hemostatic mechanism. (Error bars indicate standard error of the means, *n* = 5; ns, not significant; * indicates significant difference, ^*^ *p* < 0.05, ^**^ *p* < 0.01, ^***^ *p* < 0.001).

Hydrogel tapes serve as a protective shield for the wound, therefore must overcome a range of adverse effects associating with bacterial infection. The CMCS is an ideal solution to this problem, its amino groups trap bacteria with a negative charge via electrostatic interactions (**Figure** **3**A).^[^ ^27^ ^]^ *Escherichia coli* (gram‐negative bacterium) and *Staphylococcus aureus* (gram‐positive bacterium) are employed to evaluate the antibacterial activity of hydrogel tapes using the colony assay method (Figure 3B). ACp groups (plates b and f) do not present any antibacterial activity against *E. coli* and *S. aureus*, due to the large number of colony units on the surface of agar plates, which are similar to the control groups (plates a and e) and consistent with the quantitative analysis of bacterial viability (Figure 3C). In contrast, AC/CMCS (plates c and g) and ACAN groups (plates d and h) clearly show antibacterial activity to *E. coli* and *S. aureus*, with a bacterial inhabitation rate over 97%.

Some antibacterial chemicals (e.g., NH_4_^+^, Ag^+^, and Cu^2+^) have been known for potential cytotoxicity.^[^ ^28^ ^]^ Fortunately, the desirable configuration of CMCS chain with abundant hydroxyl, amino, and carboxyl groups keeps a balance between biocompatibility and antibacterial activity (Figure 3A).^[^ ^29^ ^]^ Fibroblasts are cultured on hydrogel tapes made of ACp, AC/CMCS, and ACAN. As shown in Figure 3D, almost all NIH‐3T3 fibroblast cells turned green after incubating for 72 h. Next, we utilize the cell counting kit‐8 (CCK‐8) assay to quantify cell viability after 24 and 72 h of incubation (Figure 3E). The cell viability for all samples is over 92%, with no explicit difference can be justified between the hydrogel treatment and control groups, which proves good cytocompatibility for hydrogel tapes.

During the wound healing, hemostasis is a critical variable.^[^ ^3^, ^30^ ^]^ A rat tail amputation hemostatic model is used to assess the hemostatic capability of hydrogel tape (Figure 3F). The untreated group serves as a control group while the other groups are treated with medical gauze, AC/CMCS, and ACAN tapes. In Figure 3G, for the blank control group, a massive amount of blood flow from the cut surface of tail covers a large area of the filter paper. Meanwhile, medical gauze absorbs copious amounts of blood from the tail without achieving hemostasis. Moreover, we observe no obvious difference in blood loss (>200 mg) and hemostasis time (>80 s) between these two groups (Figure 3H,I). There is a little trace of blood from the tail and almost no flow onto the filter paper for the AC/CMCS and ACAN groups. Notably, ACAN tape enable ultrafast hemostasis in 7 s with a blood loss of ≈28 mg, a significant improvement in hemostatic capability over gauze (*p* < 0.001).

We then perform a blood clotting index (BCI) test to evaluate the coagulation ability of hydrogel tapes using the whole blood. Compared with the AC/CMCS and ACAN‐treated groups (Figure S6, Supporting Information, BCI value ≈20%, *p* < 0.01), the gauze‐treated group had higher BCI values (>83%) after incubation. As a lower BCI value implies a better coagulation capability, the CMCS polymer chain in the dual cross‐linked structure of AC/CMCS and ACAN hydrogels provides positively charged amino groups, to generate electrostatic interactions with blood cells thus leading to adhesion and aggregation,^[^ ^31^ ^]^ when compared with the ACp hydrogel tapes (*p* < 0.05). In addition, the potential reaction between NHS ester and amine moieties in the ACAN results in moderate higher BCI (≈23.7%) than that of AC/CMCS (≈20.2%, *p* = 0.062). Massive amount of blood cells attach to the surfaces of AC/CMCS and ACAN tapes, whereas blood cells are barely visible on the surface of the gauze (Figure 3J).

When hydrogel tapes are applied upon wound with gentle pressure (Figure 3K), they absorb blood rapidly and strongly adhere to the tissue surface. Further blood coagulation is enabled via the electrostatic interactions provided by amino groups in CMCS and potential chemical reactions caused by NHS ester with cells.^[^ ^18^, ^30^ ^]^ The above hemostatic capability assessment, combined with the burst pressure test in adhesion performance evaluation, suggests a huge potential for ACAN hydrogel tape to be used in high‐pressure hemostatic applications.

### 2.4 Wound Healing Performance

**Figure 4:**
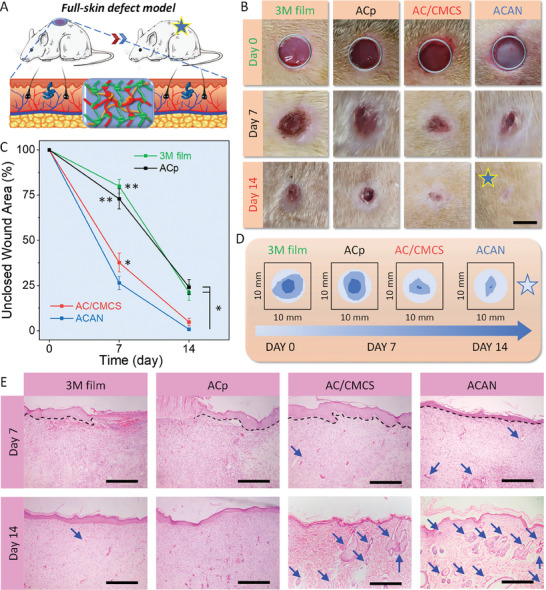
Evaluation of the wound healing performance. A) Schematic of a rat full‐skin defect model. B) Representative images the wound with different types of treatment (scale bar: 5 mm). C) Statistical analysis of unclosed wound area at different times. D) Illustration of wound area stacking from image B within 14 days of various treatments. E) Hematoxylin and eosin staining of the tissues surrounding the defects treated with 3 M film and hydrogel tapes (scale bar: 500 µm, black dashed lines indicate epithelium–dermis boundary, blue arrows indicate sebaceous glands and hair follicles. (Error bars indicate standard error of the means, *n* = 5; ns, not significant; * indicates significant difference, ^*^ *p* < 0.05, ^**^ *p* < 0.01, ^***^ *p* < 0.001).

The full‐skin defects assessment is conducted to evaluate wound healing performance (**Figure** **4**A), where the wounds are treated with commercially available 3 M film, ACp, AC/CMCS, and ACAN groups. In Figure 4B, explicit reductions in the exposed area are observed for all samples after treatment for 7 days. By treating for another 7 days, the ACAN‐treated group demonstrates a complete healing with no visible scar left. The unclosed wound area is calculated using wound area stacking to quantify the healing ability (Figure 4C,D). Following 7 days of cultivation, the ACAN‐treated group possesses an unclosed wound area of 26.4% ± 3.6%, which is much smaller than that of 3 M film (79.8% ± 3.9%, *p* < 0.01), ACp (72.9% ± 5.6%, *p* < 0.01), and AC/CMCS‐treated (37.7% ± 5.2%, *p* < 0.05) groups. After 14 days of treatment, the unclosed wound area remains at 21.1% ± 4.4% for 3 M film (*p* < 0.01) and 24.1% ± 4.3% for ACp (*p* < 0.01). Furthermore, an unclosed wound area of ≈4.5% is achieved by using AC/CMCS hydrogel, with no significant difference to ACAN. Clearly, the CMCS accelerates the wound healing process via the hydrogel tape.^[^ ^32^ ^]^

Histological analysis is carried out to understand the wound healing process by examining the neotissue surrounding the wound and site treated with 3 M film, ACp, AC/CMCS, and ACAN hydrogels. On the 7^th^ day (Figure 4E), although no inflammation is observed, some blood exudates appear from the wound sites without a complete boundary of epithelium and dermis treated with the 3 M and ACp groups. In contrast, benefiting from the cell affinity in the hydrogel, fibroblasts appear in the neotissues after treatment with AC/CMCS and ACAN hydrogels. Then, an entire epithelium layer is formed, with a distinct boundary between the epithelium and dermis (black dashed lines in Figure 4E) observed in the AC/CMCS and ACAN‐treated groups. Furthermore, only a few sebaceous glands and hair follicles are observed in these two groups (blue arrows). After 14 days of cultivation, the neotissues show basic epidermal layers in the 3 M and ACp groups, while significantly thinner epidermal layers are unveiled in AC/CMCS and ACAN‐treated groups with large number of sebaceous glands and hair follicles, demonstrating an effective wound healing.

**Figure 5:**
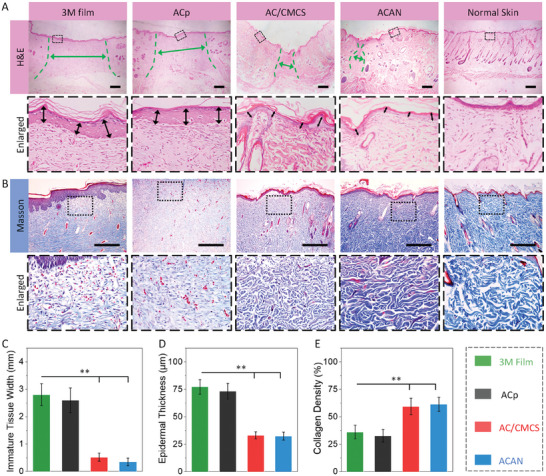
Evaluation for A) hematoxylin and eosin (H&E) and B) Masson's trichrome staining for granulation tissue sections gathered at day 14. The below columns are enlarged images of the rectangular marked area in the upper columns. Statistical analysis for C) immature tissue width, D) epidermal thickness, and E) collagen density of neotissue harvested on day 14 (scale bar: 500 µm, olive green bidirectional arrows and dashed lines indicate immature wound area, black bidirectional arrows indicate epidermal. Error bars indicate standard error of the means, *n* = 4; ns, not significant; * indicates significant difference, ^*^ *p* < 0.05, ^**^ *p* < 0.01, ^***^ *p* < 0.001).

The hematoxylin and eosin (H&E) staining for granulation tissue is assessed (**Figure** **5**A) to visualize the width of immature tissue (olive green bidirectional arrows and dashed lines) and epidermal thickness (black bidirectional arrows).^[^ ^33^ ^]^ After 14 days treatment, the immature tissue widths of AC/CMCS and ACAN groups are 0.52 ± 0.15 and 0.35 ± 0.14 mm, respectively (Figure 5C,D), which are narrower than those for 3 M film (2.8 ± 0.40 mm) and ACp (2.6 ± 0.45 mm) groups (*p* < 0.01). With an extended healing period, the epidermis becomes more uniform and thinner, resembling the structure of normal skin.^[^ ^34^ ^]^ The epidermal thickness of neotissue surrounding wound sites treated with 3 M film and ACp is 77.2 ± 6.6 and 73.3 ± 7.1 µm respectively. In contrast, they are significantly thinner (≈32.4 µm) than those ones treated by AC/CMCS and ACAN hydrogels (*p* < 0.01).

Collagen fibers play a crucial role in the growth of neotissues, whose content and distribution are key indicators for assessing wound healing.^[^ ^35^ ^]^ Compared with the 3 M film and ACp groups (Figure 5B), numerous collagen fibers can be observed in AC/CMCS and ACAN groups, as well as an increase in sebaceous glands and hair follicles. The organization of collagen fibers appears denser and more ordered after treatment with AC/CMCS and ACAN hydrogels. Furthermore, the calculated collagen volume fraction^[^ ^16^, ^36^ ^]^ results suggest that the collagen density of neotissues treated with AC/CMCS and ACAN hydrogels (≈60%) is significantly higher than these ones treated with 3 M film (36.1% ± 6.2%, *p* < 0.01) and ACp hydrogel (32.6 ± 5.7%, *p* < 0.01, Figure 5E). Hence, the higher collagen volume fraction endows a promotive healing ability to AC/CMCS and ACAN hydrogel tapes, making them more therapeutically effective than commercially available 3 M film. However, the collagen fibers triggered by inflammation wound may also lead to the formation of scar tissue, it would be interesting to investigate the mechanism of collagen deposition for scarless wound healing in the future.

## 3 Conclusions

In summary, we describe a dual cross‐linking cellulose/chitosan ACAN hydrogel with robust adhesion to wet tissue and other intriguing features, such as antibacterial, hemostasis, and effective wound healing capabilities. The dual cross‐linking structure endows ACAN hydrogel with excellent tissue‐adaptive mechanical performance and time‐dependent adhesion. The tissue adhesive tests demonstrate that the ACAN hydrogel possesses robust shear strength (≈80.8 kPa), interfacial toughness (≈170.1 J m^−2^), and high burst pressure tolerance (≈218.2 mm Hg), which significantly surpassed commercially available fibrin glue. Moreover, the inherent nature imparted ACAN hydrogel display a balance between antibacterial activity and cytocompatibility, which potentially reduces the risk of antibiotic abuse. Meanwhile, ACAN hydrogel shows better hemostatic performance than medical gauze owing to the robust adhesion to tissues and coagulation effect. The full‐skin defect model wound tests prove that ACAN hydrogel has significantly better healing ability than commercially available 3 M film concerning unclosed wound area, immature tissue width, epidermal thickness, and collagen density of neotissue.

## 4 Experimental Section

### Materials

AC, CMCS, and AA‐NHS were prepared according to previous reports.^[^ ^15^, ^27^, ^37^ ^]^ Briefly, AC was obtained by reacting the hydroxyl group on cellulose with allyl glycidyl ether to introduce functional groups of unsaturated double bonds onto the glucose unit skeleton. Similar to the reaction mechanism described above, CMCS was synthesized by substituting monochloroacetic acid for the hydroxyl group on the *O*‐position at C3/C6 and the *N*‐position of chitosan, resulting in an amphoteric ether derivative with carboxyl and amino groups. The detailed preparation procedures and other required chemicals are presented in Supporting Information.

### Fabrication of the Hydrogel Tapes

For prefabrication of hydrogel tapes (Figure 1A), three types of precursor hydrogel solutions were prepared in PBS solution (pH = 7.4) at room temperature: ACp contained 4.0% (w/v) allyl cellulose and 0.2% (w/v) LAP. AC/CMCS comprised an ACp precursor solution and an additional 4.0% (w/v) CMCS. ACAN comprised the AC/CMCS precursor solution and 1.5% (w/v) AA‐NHS. The composition of hydrogel tapes are listed in Table S1, Supporting Information. All precursor solutions were then poured into a glass mold with spacers and cured under a blue light source (405 nm, 25 mW cm^−2^) for 20 min. The as‐obtained hydrogel tapes were dried in air until the water content was 50% ± 2.5% with thickness of 0.78 ± 0.08 mm.

### Characterization

The obtained AC and CMCS samples with concentration of 1% (w/v) in D_2_O and AA‐NHS with the same concentration in DMSO‐*d_6_* were characterized using a Bruker instrument (AVANCE III, Germany) under 400 MHz to record ^1^H NMR spectra. Attenuated total reflection‐Fourier transform infrared spectroscopy (ATR‐FTIR) instrument (Bruker VERTEX 70, Germany) was used to characterize chemical composition of AC, CMCS AA‐NHS, and ACAN hydrogel. An SEM (JSM‐5600 V, Japan) was used to observe the surface morphology. The samples to be characterized had to be freeze‐dried and dissected prior to test.

### Mechanical Performance

The tensile and unconfined compression tests were performed using an INSTRON instrument (3382, Norwood, USA) at 25 °C and a relative humidity of 50%. The samples were prepared as a cylinder with a diameter and height of 15 mm for the compression test and a dog bone shape with a length of 10 mm, thickness of 1 mm, and width of 2 mm for the tensile test. At a speed of 2 mm min^−1^, samples were tested and repeated five times, and then, stress–strain curves for tensile and compression were recorded using real‐time monitoring. The moduli were calculated using the slope of the obtained curve during the elastic deformation phase (0%–20%). The work of fracture was calculated using the area enclosed within the stress–strain curve and the base.^[^ ^38^ ^]^

### Swelling Ratio and In Vitro Enzymatic Degradation Tests

Briefly, the ACp, AC/CMCS, and ACAN samples were incubated in PBS at 37 °C for 48 h, and the swelling ratio was calculated by dividing the samples’ dry weight to the swollen weight. The degradation tests were performed using enzymatic degradation media based on a previous study with a modification procedure.^[^ ^24^ ^]^ Briefly, ACp, AC/CMCS, and ACAN samples were prepared as cylindrical shapes for the degradation test in PBS containing 2 mg mL^−1^ lysozyme. The degradation rate was defined and calculated by the ratio of initial dry weight to residual weight after degradation. More details are available in Supporting Information.

### Tissue Adhesive and Burst Pressure Tests

The shear strength of ACp, AC/CMCS, and ACAN samples was measured using porcine skin as a tissue model via a lap shear test with an INSTRON instrument (3382, Norwood, USA), and the adhesion area was 1 cm in width and 1.5 cm in length. All specimens were tested at 25 °C with a relative humidity of 50% and a strain speed of 2 mm min^−1^. The shear strength was defined as the maximum stress divided by the adhesion area. The poly(methyl methacrylate) (PMMA) films were bonded using SUPER GLUE (Pattex).

To gauge tensile strength of hydrogel tapes, adhered samples with a square adhesion area of 4 cm^2^ were manufactured and fixed to a homemade aluminum holder (Figure S5A, Supporting Information). The tests were performed with an INSTRON instrument (3382, Norwood, USA) based on a standard (ASTM F2258). The interfacial toughness of hydrogel tapes was evaluated using a 180° peel test according to the standard (ASTM F2256).^[^ ^2^ ^]^ As shown in the right half of Figure S5A, Supporting Information, PMMA backing was utilized for adhesion to porcine skin tissues with a width of 1 cm and tested with the INSTRON‐3382 instrument.

To evaluate the sealing ability of hydrogel tapes under a specific pressure range, burst pressure test was performed based on the methods and standard (ASTM F2392).^[^ ^39^ ^]^ Figure S5D (Supporting Information) shows a customized testing setup, which primarily comprised a peristaltic pump (LEAD FLUID, BT100S) and a pressure gauge (Haoglobe, PM‐200). First, a piece of moist porcine skin tissue (22 mm in diameter and 4 mm in thickness) with a 2 mm defect was fixed on a glass tube connected to a peristaltic pump, and then hydrogel tapes were applied to cover the surface of porcine skin. Filling PBS at a rate of 2 mL min^−1^ was used to continually increase the pressure, and a pressure gauge was used to read the pressure via real‐time recording.

### In Vitro Antibacterial Activity and Cytocompatibility Tests

*Escherichia coli* (ATCC 25 922, gram‐negative bacterium) and *S. aureus* (ATCC 6538, gram‐positive bacterium) were employed to evaluate the antibacterial activity. ACp, AC/CMCS, and ACAN hydrogels were selected as the experimental groups. And the specimens without hydrogel were set as control group. Briefly, the pre‐prepared suspension (10^6^ CFU mL^−1^, 0.01 mL) and 0.99 mL PBS were added into centrifuge tube, then sterilized ACp, AC/CMCS, and ACAN hydrogel tapes were added separately. After incubation and dilution, the number of colony‐forming units (CFUs) was counted, and the agar plates were photographed.^[^ ^40^ ^]^ More details can be found in Supporting Information.

NIH‐3T3 fibroblasts (SCSP‐515, Stem Cell Bank, Chinese Academy of Sciences, Shanghai, China) were used to assess cytocompatibility and cultured on the ACp, AC/CMCS, and ACAN hydrogels, following a previous study.^[^ ^25^ ^]^ Concisely, before cell seeding, the hydrogels with diameter of 5 mm and thickness of 2 mm were first purified in PBS and sterilized under UV lamp for 30 min. Subsequently, the hydrogels were immersed in Dulbecco minimum essential medium (DMEM, HyClone, USA) to allow a swelling equilibrium state. CCK‐8 assay was employed to quantitatively evaluate the cell viability. To visualise cell viability, live/dead staining method was used for cell imaging by calcein‐AM/propidium iodide (PI) staining. To evaluate the cytotoxicity, five parallel specimens for each group were tested. Additional details are provided in Supporting Information.

### Hemostatic Capability Test

The hemostatic ability of hydrogel tapes was determined using a rat tail amputation model. Briefly, the same number of Sprague Dawley (SD) male rats in each group had 30% of their tail length cut off and exposed to air for 15 s before testing, followed by rapidly wrapping the wounds with medical gauze, AC/CMCS, and ACAN tapes. Hemostatic ability was assessed by recording the amount and duration of blood loss.

To evaluate the effect of blood coagulation, whole blood clotting tests were performed to examine the BCI values. Furthermore, SEM was employed to observe and analyze the morphology of blood cells attached on medical gauze and ACAN hydrogel tape. More details are available in Supporting Information.

### In Vivo Wound Healing Test

For full‐skin tests, all SD male rats (385–415 g, 8 weeks) were housed for 1 week before surgery. The biopsy punch was used to create four full‐thickness circular skin wounds with a diameter of 7 mm on rats under aseptic conditions. The AC, AC/CMCS, and ACAN hydrogel tapes were the experimental groups and the 3 M film (3 M Health Care, USA) was set as the control group. The experiments were conducted on every five SD rats in the four groups. The wound areas were measured and objectively analyzed using Image‐Pro Plus 6.0 software.

One centimeter square tissue specimens collected at different intervals were used for the histological and collagen density analysis. The paraformaldehyde‐fixed specimens were sectioned to a thickness of 40 µm with H&E and Masson's trichrome staining and evaluated under a microscope. More details are described in Supporting Information.

### Animal Ethics Statement

All animal experiments were performed following the protocol approved by the ethics board of school of chemical engineering, Nanjing Forestry University, China. (Ethic Approval Ref: ACAN) and in strict accordance with the ARRIVE guidelines 2.0.

### Statistical Analysis

All data assessed statistically using Origin Pro 2016 software were described as mean ± standard deviation. One‐way ANOVA was utilized to determine differences between mean values. Mean differences were considered statistically significant if the confidence interval exceeded 95% (* indicated significant difference, ^*^ *p* < 0.05, ^**^ *p* < 0.01, ^***^ *p* < 0.001).

## Conflict of Interest

The authors declare no conflict of interest.

## Supporting information

Supporting Information

## Data Availability

The data that support the findings of this study are available in the Supporting Information material of this article.
