# Supplementary material for: A Cellulose/Chitosan Dual Cross‐Linked Multifunctional and Resilient Hydrogel for Emergent Open Wound Management
Source: Adv Healthc Mater. 2024 Feb 13;13(13):2304676. doi: 10.1002/adhm.202304676 (PMC11468647; doi:10.1002/adhm.202304676)
Supplement: Supplementary file 1 — Supporting Information [file ADHM-13-2304676-s001.pdf]

ADVANCED HEALTHCARE MATERIALS

Supporting Information

for *Adv. Healthcare Mater.*, DOI 10.1002/adhm.202304676

A Cellulose/Chitosan Dual Cross-Linked Multifunctional and Resilient Hydrogel for Emergent Open Wound Management

Shengchang Lu, Hui Wu, Shengbo Ge, Liulian Huang, Lihui Chen*, Chris Connor, Zhanhu Guo, Yunhong Jiang, Ben Bin Xu* and Wanxi Peng**

Supporting Information

for

A Cellulose/Chitosan Dual-Crosslinked Multifunctional and Resilient Hydrogel for Emergent Open Wound Management

Shengchang Lu[1,2,3,#], Hui Wu[2,3,#], Shengbo Ge[4,*], Liulian Huang[2,3], Lihui Chen[2,3,*], Chris Connor[5], Zhanhu Guo[5], Yunhong Jiang[6], Ben Bin Xu[5,*], Wanxi Peng[1,*]

[1] *School of Forestry, Henan Agricultural University, Zhengzhou 450002, P. R. China*

[2] *College of Material Engineering, Fujian Agriculture and Forestry University, Fuzhou, Fujian 350002, P. R. China*

[3] *National Forestry and Grassland Administration Key Laboratory of Plant Fiber Functional Materials, Fuzhou, Fujian 350002, P. R. China*

[4] *Co-Innovation Center of Efficient Processing and Utilization of Forest Resources, College of Materials Science and Engineering, Nanjing Forestry University, Nanjing 210037, China*

[5] *Mechanical and Construction Engineering, Northumbria University, Newcastle Upon Tyne NE1 8ST, UK*

[6] *Hub for Biotechnology in the Built Environment, Department of Applied Sciences, Northumbria University, Newcastle upon Tyne, NE1 8ST, UK*

* Corresponding authors.

E-mail addresses: geshengbo@njfu.edu.cn (S. Ge); fafuclh@163.com (L. Chen); ben.xu@northumbria.ac.uk (B.B. Xu); pengwanxi@163.com (W. Peng)

[#] These authors contributed equally to this work.

Experimental Section

Materials: Cellulose was extracted from a pinus massoniana dissolving pulp board, supplied by Fujian Qingshan Paper Industry Co., Ltd., China. Allyl glycidyl ether (AGE, 99%), chitosan (CS, degree of deacetylation ≥ 95%), isopropanol (ACS, ≥ 99.5%), N-Hydroxysuccinimide (NHS, 98%) and triethylamine (TEA, 99.5%) were purchased from Beijing InnoChem Science & Technology Co., Ltd, China. Sodium hydroxide (NaOH), ethanol (AR, 99.7%), acetone, hydrochloric acid, urea, tetrahydrofuran (THF), agar and phosphate buffer saline (PBS) were provided by Shanghai Aladdin Reagent Co., Ltd, China. Acryloyl chloride (Ac, 96%) and monochloroacetic acid (ACS, ≥ 99%) and 2,4,6-trinitrobenzene sulfonic acid (TNBS, 5% in water) were purchased from Sigma-Aldrich (St. Louis, MO, USA). The blue light source (LS-1601, 405nm) and photo-initiator lithium phenyl-2,4,6-trimethylbenzoylphosphinate (LAP) were purchased from Suzhou Intelligent Manufacturing Research Institute (Suzhou, China). All chemicals were used as received. Peptone, yeast powder, lysozyme, *Escherichia coli* (*E. coli*, ATCC 25922) and *Staphylococcus aureus* (*S. aureus*, ATCC 6538) were supplied by Shanghai Bioresource Collection Center. Ultrapure water (18.2 MΩ·cm) obtained by a water purifier (Sichuan Water Purifier Co. Ltd., China) was used for all experiments.

Synthesis of allyl cellulose (AC): As shown in **Figure S1A**, AC was synthesized according to previous reports[1]. Briefly, AGE (10 times molar ratio to anhydroglucose unit of cellulose) was added dropwise into an aqueous NaOH/urea solution with cellulose (4.0 wt%), and then reacted under stirring with N_2 flow at 30 °C for 24 h. By a general procedure of precipitation in acetone, dialysis and freeze-drying, a white foamy AC was obtained. ^{1}H NMR spectra was conducted to confirm the existence of unsaturated ethylenic bonds (peaks around 5.63 and 4.96 ppm) in allyl cellulose, and the substitution degree was calculated to be 2.45 by integration of the signals arising from the α anomeric proton of glucose residues (peak at 4.43 ppm) and the ethylenic protons.

Synthesis of carboxymethyl chitosan (CMCS): As shown in **Figure S1B**, CMCS was synthesized according to previous study[2]. Briefly, after dissolution of chitosan powder (5.0 g) in isopropanol (120 mL), 35 mL of sodium hydroxide (68 wt%) in water/isopropanol (1:1 V/V) solution was added. Subsequently, 24 g of monochloroacetic acid/isopropanol solution (1:1 m/m) was added dropwise to the mixture to react for 5 hours. After being washed by ethanol/water mixture and neutralised with

hydrochloric acid, the sample was obtained under vacuum drying. ^{1}H NMR spectra was employed to characterise the structure of prepared CMCS. Specifically, chemical shifts between 4.55 and 4.75 ppm are the H-1 of chitosan resonance. The peak at 2.68 is H-2 proton resonance. The chemical shifts between 4.05 and 4.35 ppm are the protons of C3 and C6-substituted carboxymethyl (-O-CH_2-COO-) of CMCS, respectively. Furthermore, the content of the primary amino group in CMCS was 0.63 ± 0.06 mmol/g by the TNBS method[3]. The degree of carboxymethyl substitution of CMCS was 0.61, which was calculated by the ^{1}H NMR spectra based on the method in previous study[4].

Synthesis of acrylic acid *N*-hydroxysuccinimide (AA-NHS): To synthesize AA-NHS (**Figure S1C**), 10.0 g of NHS (86.9 mmol) and 15 mL of TEA were dissolved in 150 mL of chloroform in an ice-water bath, and 8.5 mL of Ac was slowly added dropwise to the solution. At the end of the reaction, the solution was washed twice with ice water and saturated sodium chloride solution. Then the organic phase was separated and dried with $MgSO_4$, and the solution was then filtered and further concentrated to remove the chloroform. The concentrate was further recrystallised in hexane/ethyl acetate (8:1) and the crystals were filtered and washed with hexane/ethyl acetate to obtain a white precipitate, which was dried under vacuum for 24 h to give a purified AA-NHS with yield of 80.2%. As shown in **Figure S1C**, the signal (6.65-6.71, 6.31-6.36 ppm, 2H) was attributed to the H peak of the unsaturated bonds -CH=C$\underline{\boldsymbol{H}}_2$. The signal (6.47-6.55 ppm, 1H) was attributed to the H peak of -C$\underline{\boldsymbol{H}}$=$CH_2$. The signal (2.84 ppm, 4H) was assigned to the H peak of the succinimide framework. Furthermore, the ratio of peak area integration (2.02:1.06:4.11) at all the above positions is in accordance with the theoretical value (2:1:4)[5].

Swelling ratio of the hydrogel tapes: The ACp, AC/CMCS, and ACAN samples were incubated in PBS at 37 °C for 48 h, then lightly blotted dry and weighed (W_s). Hydrogels were then freeze-dried and weighed to determine the dry weight (W_d). The tests were performed 4 times. The swelling ratio of the swollen samples were calculated via the following equation:

$$\text{Swelling ratio} = \frac{W_S - W_d}{W_d} \times 100\%$$

In vitro enzymatic degradation: The tests of hydrogel tapes were carried using enzymatic degradation media as displayed in previous study [6]. Briefly, ACp, AC/CMCS, and ACAN samples

were prepared as cylindrical shape with a diameter of 5 mm and height of 2 mm for the degradation test. The samples were firstly lyophilized and weighted (W_i). Then they were fully immersed in 5 mL phosphate buffered saline containing 2 mg/mL lysozyme at 37°C. Fresh medium was replaced every two days with the same lysozyme concentration. The samples were weighed at the designed time intervals and weighed as W_t. The degradation rate (100%) was calculated from the mass loss and the residual mass ratio was calculated by dividing W_t. The tests were performed at 37°C and 60 r/min on a constant temperature shaker, using four replicates to assess in vitro enzymatic degradability.

Determination of the degree of crosslinking: The solid contents of hydrogels before and after blue light irradiation were weighted to measure the degree of crosslinking according to the reported method with some modification[7]. The as-prepared hydrogels were immersed in PBS for 12 hours to remove the uncross-linked reactants. The hydrogels before and after immersion in water was freeze-dried and weighted. The original dry weight of the hydrogel before immersion could be defined as W_o and the dry weight of the cross-linked hydrogel after immersion could be defined as W_c. The degree of crosslinking (D_c %) was calculated using the following equation:

$$D_c = \frac{W_c}{W_o} \times 100\%$$

All measurements were obtained by repeating the experiment three times.

In vitro antibacterial activity: *E. coli* (ATCC 12228, Gram-negative) and *S. aureus* (ATCC 6538, Gram-positive) were used to evaluate the antibacterial activity of the samples. During antibacterial tests, the ACp, AC/CMCS and ACAN hydrogels were selected as the experimental groups. And the specimens without hydrogel were set as blank group. The obtained *E. coli* and *S. aureus* suspensions (10^8 CFU/mL) were deposited in the sterile medical bottles. The solution was then diluted 100 times further to obtain a working suspension (10^6 CFU/mL), then the suspensions (0.01 mL) with PBS (0.99 mL) were added into the centrifuge tubes, followed by the addition of ACp, AC/CMCS and ACAN samples, respectively. The centrifuge tubes were then incubated at 37 °C for 24 h with 200 rpm in a shaker. The diluted bacterial solution (100 times) was taken out and plated on Luria-Bertani (LB) agar for 24 h at 37 °C. The colony forming units (CFUs) on the petri dishes were counted and the bacterial growth on each agar plate was photographed[8]. The antibacterial test was performed on five samples from each group in parallel.

Biocompatibility test: NIH-3T3 fibroblasts (SCSP-515, Stem Cell Bank, Chinese Academy of Sciences, Shanghai, China) were cultured on the ACp, AC/CMCS and ACAN hydrogels[9]. Briefly, before cell seeding, the hydrogels with diameter of 5 mm and thickness of 2 mm were first purified in PBS and sterilized under UV lamp for 30 min. Subsequently, the hydrogels were immersed in Dulbecco minimum essential medium (DMEM, HyClone, USA) to allow a swelling equilibrium state and added into 48-well tissue culture plates. NIH-3T3 cells were cultured in DMEM with 10% fetal bovine serum (HyClone) and 1% penicillin-streptomycin solution (HyClone) in a CO_2 incubator at 37 °C. NIH-3T3 cell suspension (500 μL) was seeded on the hydrogels at a density of 10^4 cells/well. Then the cell-containing hydrogels were cultured in DMEM supplemented with 10% fetal bovine serum and 1% penicillin-streptomycin at 37 °C and 5% CO_2 for 24 and 72 hours. The cell growth on the hydrogels was quantified using the CCK-8 (HY-K0301, MCE) assay. The hydrogels were washed twice by PBS and CCK-8 reagent (500 μL, 10%) was added to each well and incubated for 2 h at 37 °C. And the absorbance at 450 nm was measured by a microplate reader (DR-200Bn, Diatek). The relative viability of cell was calculated by the ratio of OD value of samples and control group. Cells on the hydrogels were stained using Calcein-AM/propidium iodide (PI) (BB4126-1, BestBio, Shanghai, China). After the cells were cultured for the predetermined times, 200 μL of Calcein-AM/PI was added to the samples and incubated for 20 min in the dark at 4 °C. The cells were observed using a confocal laser scanning microscope (TCS SP8, Leica, Germany). To evaluate the cytotoxicity of the hydrogels, five parallel specimens from each group were used.

Haemostatic capability test: The hemostatic ability of the hydrogel tapes was estimated by the mouse-tail amputation model. All animal experimental procedures were performed in accordance with the protocol approved by the Animal Experimentation Ethics Committee of Fujian Agriculture and Forestry University, and strictly complied with the guideline of EU Directive 2010/63/EU. All Sprague Dawley (SD) male rats (385~415 g, 8 weeks) were randomly divided into four groups in equal number. After cutting thirty percent length of the tail and exposed in air for 15 s, the wound was quickly wrapped with gauze, AC/CMCS and ACAN tapes, respectively. The mass and time of blood loss were all recorded and five parallel specimens for each group were tested to evaluate the hemostatic performance.

To evaluate the blood coagulation effect, whole blood-clotting tests were conducted to study the blood clotting index (BCI) values of the samples. The procedure was as follows: ***1.*** Samples were pre-warmed (37 °C) in centrifuge tubes for 10 minutes. ***2.*** The whole blood of rat (0.4 mL) within sodium citrate solution (0.04 mL, 38 mg/mL) was slowly dropped on samples to cover the surface completely and $CaCl_2$ solution (0.03 mL, 0.2 M) was added for coagulation. ***3.*** After incubation at 37 °C for the designed time intervals (1, 3, and 5 minutes), 10 mL of ultrapure water was added to the mixture, then collected and centrifuged (800 r/min) for 30 seconds. The 3-fold-diluted supernatant was kept at 37 °C for 60 minutes. The relative absorbance of diluted supernatant blood specimens at 540 nm was measured by microplate reader (DR-200Bn, Diatek) to obtain I_s, and the absorbance of the whole mouse blood (0.4 mL) directly mixed with 30 mL ultrapure water was measured as I_w. The BCI values were calculated by the following equation[10]:

$$\mathrm{BCI} = \frac{I_s}{I_w} \times 100\%$$

To observe and analyse the morphology of blood cells on medical gauze and ACAN hydrogel tape, the recalcified whole blood was dripped onto the surface of samples and then incubated at 37 °C for 10 min. After removing the physically adhered blood cells on all the samples by PBS (pH 7.4), 2.5 wt.% glutaraldehyde was used to fix the samples at 37 °C for 12 h. Then, all the samples were gradually dehydrated at gradient concentrations from 60% to 99.5% of ethanol solutions and an interval of 15 min. Finally, SEM was used to observe and analyse the morphology of the freeze-dried samples.

In vivo wound healing test: All SD male rats (385~415 g, 8 weeks) were acclimatised for 7 days before surgery. The surgical procedures were performed under aseptic conditions[6, 11]. Firstly, all the rats were anaesthetised by the injection of chloral hydrate (0.3 mg/kg body weight) in the peritoneum; then, the dorsal area of rats was shaved with a razor. The biopsy punch was used to create 4 full-thickness circular skin wounds (7 mm in diameter). After the removal of wound skin, the transparent dressing 3M film (3M Health Care, USA) was used to cover the wound bed and set as the control group. The ACp, AC/CMCS, and ACAN hydrogel tapes were implanted and covered on other wound sites of the rats. All tissues were collected on every 5 rats in 4 groups at an interval of 7 days. All tissue samples were kept at -80 °C for further analysis.

The wound regeneration procedure was evaluated by wound area monitoring and biochemical and histomorphological assessment. The wound areas were measured at day 7 and 14 after the rats were anaesthetised. Then, the wound area was analysed by drawing and measuring the wound boundaries by Image-Pro Plus 6.0 software. All photographs of the wound were read blinded and the operator was unaware of the applied samples to ensure an objectivity assessment.

Histological and collagen deposition analysis: To assess the epidermal regeneration and collagen deposition in wound areas, histological analysis of specimens at different stages with 1 cm^2 square-cut was performed by Haematoxylin-Eosin (H&E) and Masson's trichrome (Beyotime Biotechnology, China) stain. Briefly, the regenerated wound tissues collected at different time intervals were fixed with 4% paraformaldehyde for 1 day. Then, the specimens were embedded into paraffin with a thickness of 40-μm cross-sectional slices. All slices were stained with H&E and Masson's trichrome stain for histological analysis and analysed under an OLYMPUS microscope (BX53M, Japan).

Table S1. Composition and degree of crosslinking of the different hydrogel tapes.

Hydrogel tape	AC (g)	CMCS (g)	AA-NHS (g)	LAP (g)	PBS (mL)	D_c (%)
ACp	0.4	/	/	0.02	10	97.2±0.4
AC/CMCS	0.4	0.4	/	0.02	10	96.8±0.3
ACAN	0.4	0.4	0.15	0.02	10	97.4±0.3

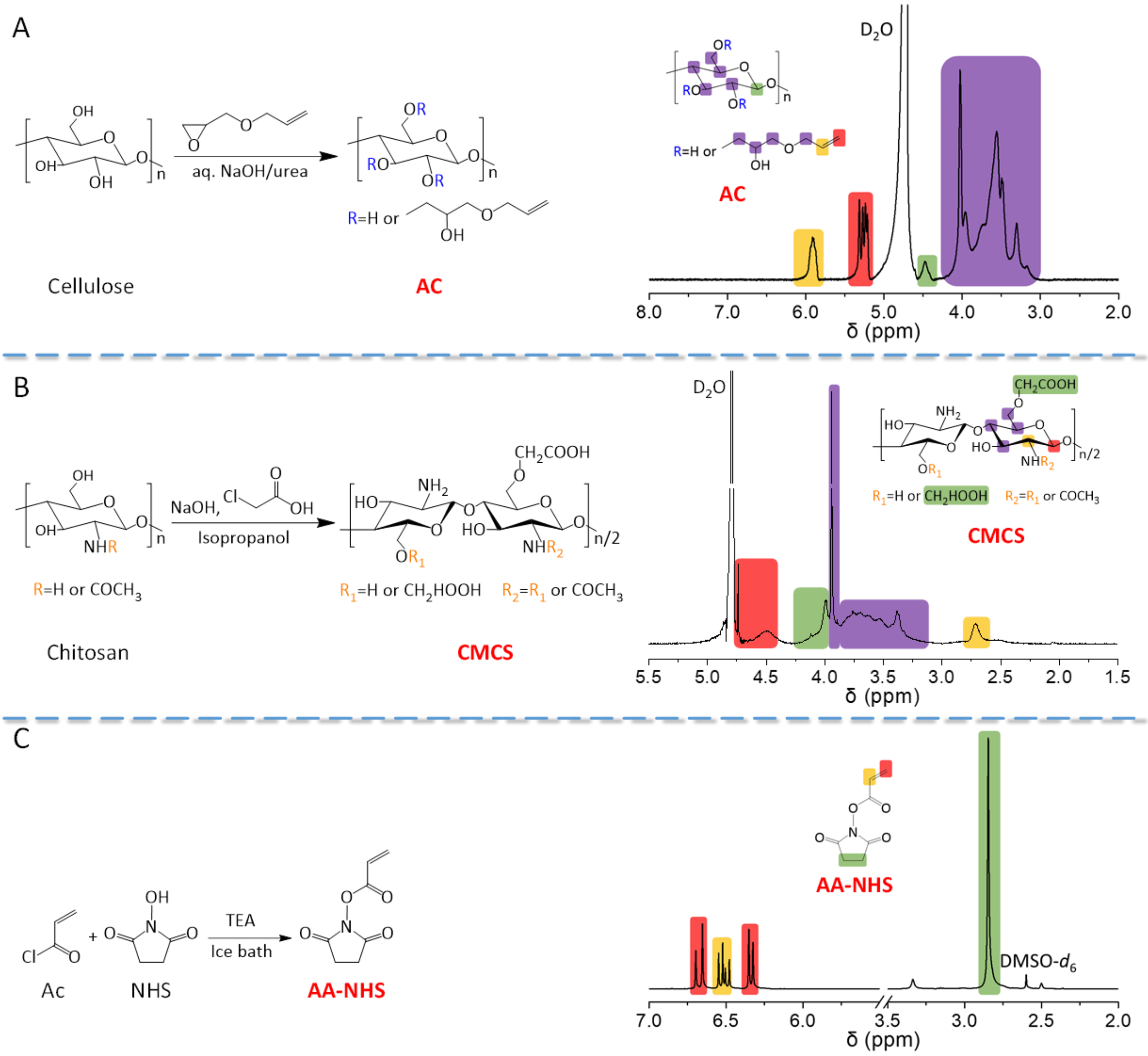

Figure S1. Synthesis routes and ^{1}H-NMR spectrums of (A) AC, (B) CMCS and (C) AA-NHS.

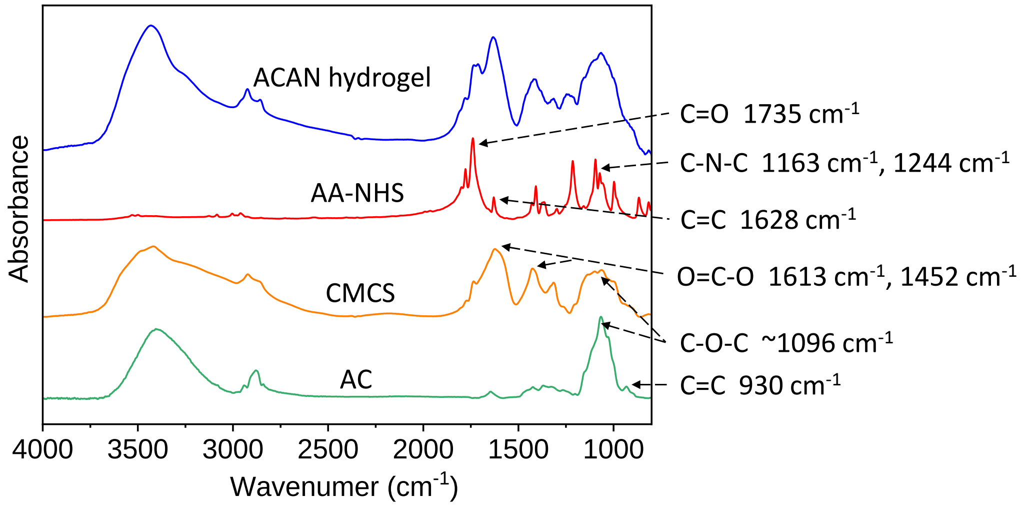

Figure S2. ATR-FTIR spectra of AC, CMCS, AA-NHS and ACAN hydrogel.

The vibrations of the C=C bond in AC moiety were detected at 930 cm^{-1} [12]. The band at around 1096 cm^{-1} was assigned to the characteristic stretching vibration of the C-O-C group presented in AC and CMCS. The characteristic peaks at 1613 cm^{-1} and 1452 cm^{-1} were attributed to the carboxylate band of CMCS [3, 13]. The peak at 1628 cm^{-1} was associated with C=C and the peak in 1735 cm^{-1} was attributed to the stretching vibrations of C=O groups of AA-NHS, the symmetric C–N–C stretch at 1163 cm^{-1} and asymmetric C–N–C stretch at 1244 cm^{-1} were associated with NHS ester [14]. Moreover, the disappearance of the characteristic peak of the unsaturated double bond and the appearance of the peaks corresponding to the NHS ester in the spectrum of ACAN hydrogel demonstrated that chemical crosslinking occurred via free radical polymerisation.

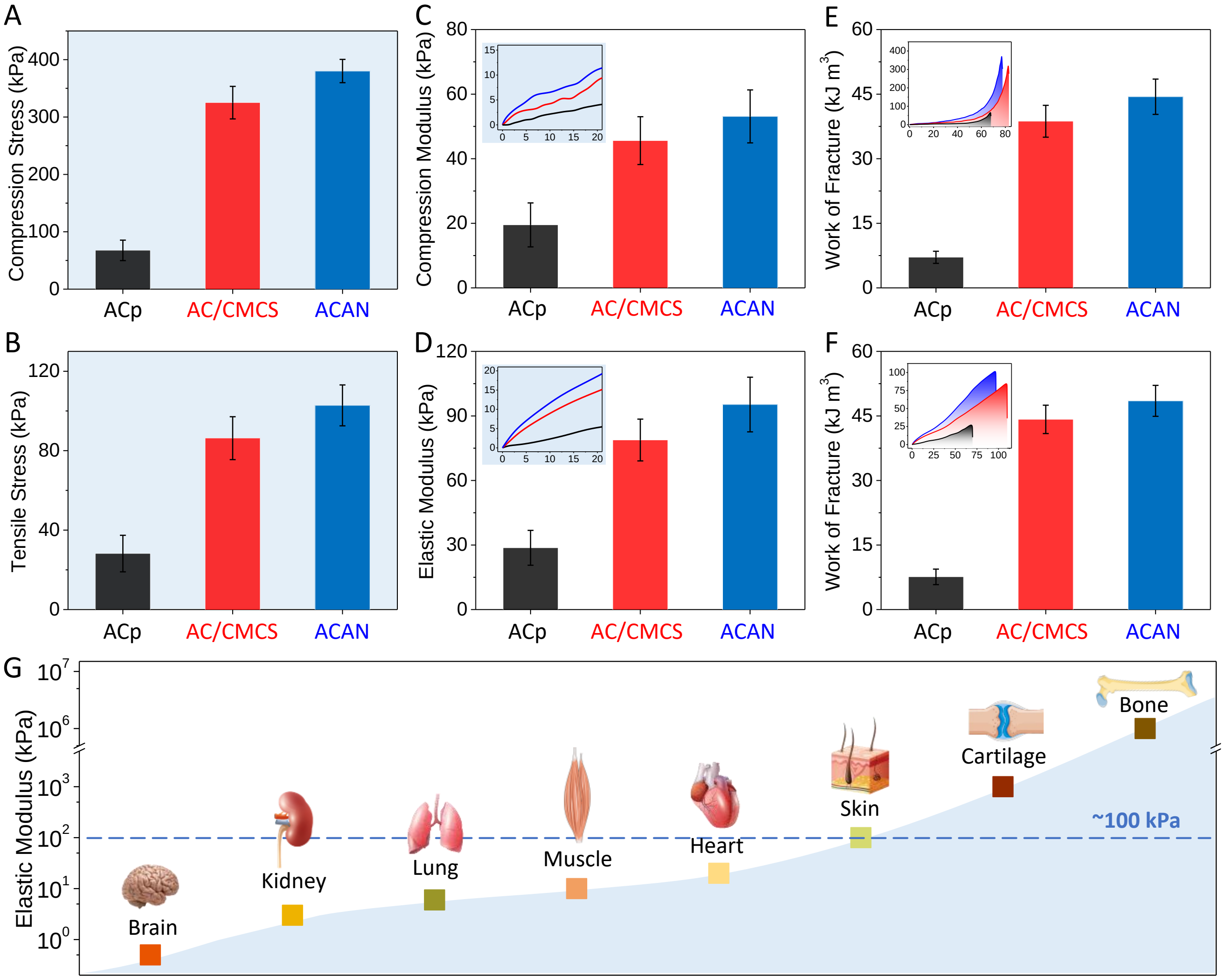

Figure S3. Maximum (A) compression and (B) tensile stress of hydrogels. (C) Compression and (D) elastic modulus of various hydrogels. Work of fracture under (E) compression and (F) tension mode of various hydrogels. (G) Elastic moduli of biological tissues[15]. (Embedded figures in C and D represents typical stress–strain curves from 0 to 20%).

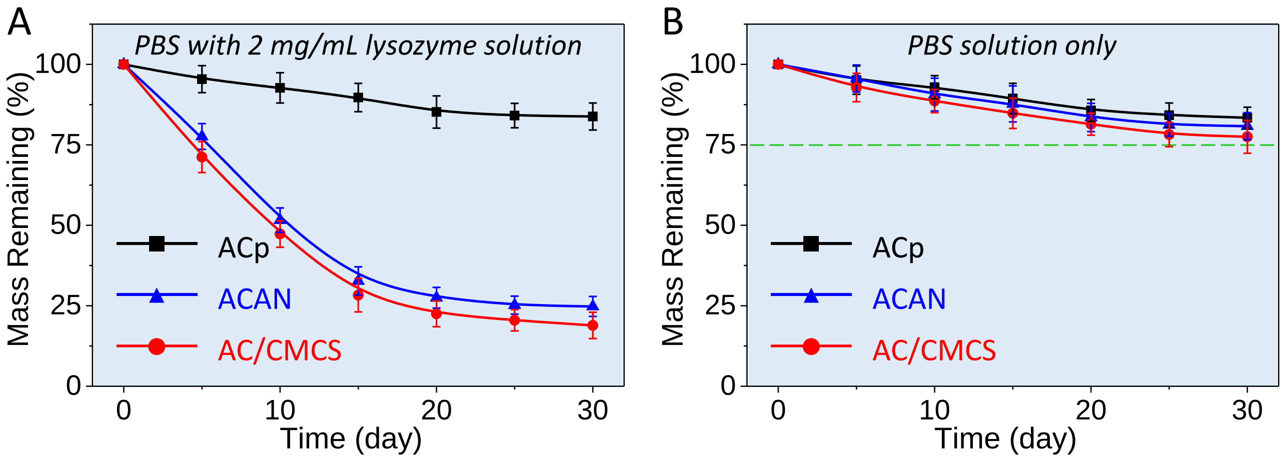

Figure S4. In vitro degradation of the hydrogels in (A) PBS with lysozyme solution and (B) PBS solution only.

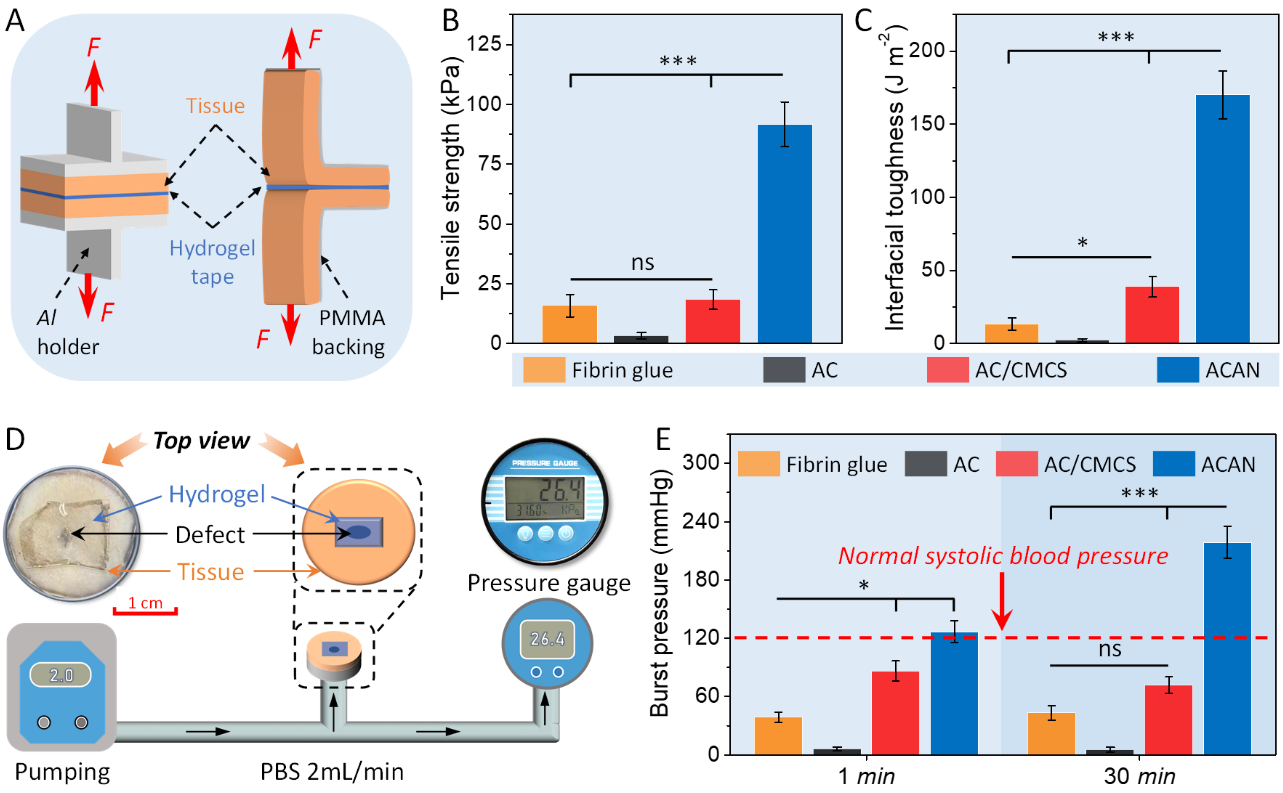

Figure S5. (A) Schematic illustration of measurement for tensile strength and interfacial toughness. (B) Tensile strength and (C) interfacial toughness for commercially-available Fibrin glue and hydrogel tapes. (D) Schematic diagram of home-made setup for burst pressure test. (E) Summary of burst pressures for commercially-available Fibrin glue and hydrogel tapes. (Error bars indicate standard error of the means, n = 5; ns, not significant; * indicates significant difference, *$p < 0.05$, **$p < 0.01$, ***$p < 0.001$).

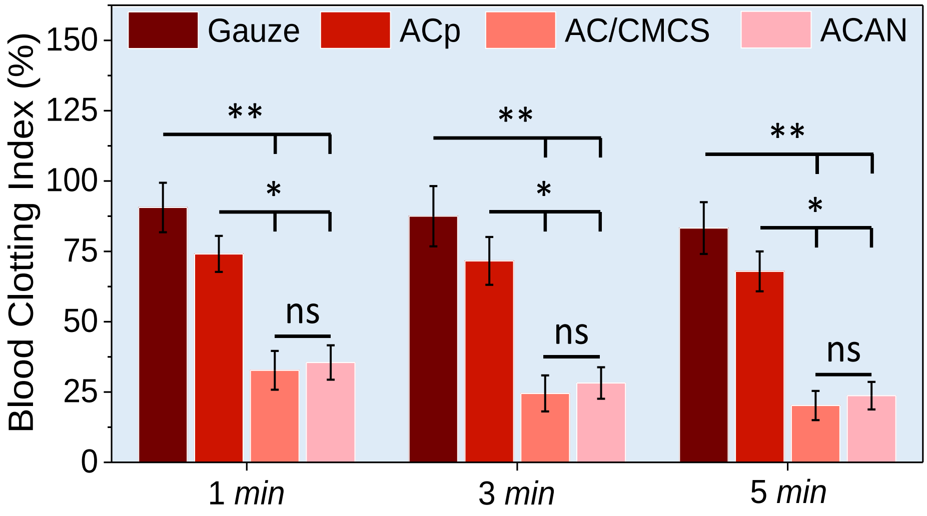

Figure S6. Blood clotting index of the gauze and hydrogel tapes in different durations. (Error bars indicate standard error of the means, n = 5; ns, not significant; * indicates significant difference, *$p < 0.05$, **$p < 0.01$, ***$p < 0.001$).

References

[1] a) R. Tong, G. Chen, D. Pan, H. Qi, R. a. Li, J. Tian, F. Lu, M. He, *Biomacromolecules* **2019**, 20, 2096; b) S. Lu, X. Zhang, Z. Tang, H. Xiao, M. Zhang, K. Liu, L. Chen, L. Huang, Y. Ni, H. Wu, *Chem. Eng. J.* **2021**, 417, 129329.

[2] Y. Geng, H. Xue, Z. Zhang, A. C. Panayi, S. Knoedler, W. Zhou, B. Mi, G. Liu, *Carbohydr. Polym.* **2023**, 305, 120555.

[3] W. Huang, Y. Wang, Z. Huang, X. Wang, L. Chen, Y. Zhang, L. Zhang, *ACS Appl. Mater. Interfaces* **2018**, 10, 41076.

[4] X.-G. Chen, H.-J. Park, *Carbohydr. Polym.* **2003**, 53, 355.

[5] A. Hasneen, I.-s. Cho, K.-W. Kim, H.-j. Paik, *Polym. Bull.* **2012**, 68, 681.

[6] Y. Ma, J. Yao, Q. Liu, T. Han, J. Zhao, X. Ma, Y. Tong, G. Jin, K. Qu, B. Li, F. Xu, *Adv. Funct. Mater.* **2020**, 30, 2001820.

[7] a) A. Bigi, G. Cojazzi, S. Panzavolta, K. Rubini, N. Roveri, *Biomaterials* **2001**, 22, 763; b) S. Bian, L. Hao, X. Qiu, J. Wu, H. Chang, G.-M. Kuang, S. Zhang, X. Hu, Y. Dai, Z. Zhou, F. Huang, C. Liu, X. Zou, W. Liu, W. W. Lu, H. Pan, X. Zhao, *Adv. Funct. Mater.* **2022**, 32, 2207741.

[8] K. Wang, J. Wang, L. Li, L. Xu, N. Feng, Y. Wang, X. Fei, J. Tian, Y. Li, *Chem. Eng. J.* **2019**, 372, 216.

[9] H. Xu, L. Zhang, J. Cai, *ACS Appl. Bio Mater.* **2019**, 2, 196.

[10] J. Zhu, H. Han, F. Li, X. Wang, J. Yu, X. Qin, D. Wu, *Chem. Mater.* **2019**, 31, 4436.

[11] L. Han, Y. Zhang, X. Lu, K. Wang, Z. Wang, H. Zhang, *ACS Appl. Mater. Interfaces* **2016**, 8, 29088.

[12] H. Qi, T. Liebert, T. Heinze, *Cellulose* **2012**, 19, 925.

[13] K. Zhang, Y. Xian, M. Li, Z. Pan, Z. Zhu, Y. Yang, H. Wang, L. Zhang, C. Zhang, D. Wu, *Adv. Funct. Mater.* **2023**, 33, 2305222.

[14] a) J. He, Z. Zhang, Y. Yang, F. Ren, J. Li, S. Zhu, F. Ma, R. Wu, Y. Lv, G. He, B. Guo, D. Chu, *Nano-Micro Lett.* **2021**, 13, 80; b) K. Zhang, X. Chen, Y. Xue, J. Lin, X. Liang, J. Zhang, J. Zhang, G. Chen, C. Cai, J. Liu, *Adv. Funct. Mater.* **2022**, 32, 2111465.

[15] S. Nam, D. Mooney, *Chem. Rev.* **2021**, 121, 11336.
